# Recent Advances in the Genetics of Systemic Sclerosis: Toward Biological and Clinical Significance

**DOI:** 10.1007/s11926-014-0484-x

**Published:** 2015-03-18

**Authors:** Benjamin D. Korman, Lindsey A. Criswell

**Affiliations:** 1 Division of Rheumatology, Feinberg School of Medicine, Northwestern University, Chicago, IL USA; 2 Rosalind Russell/Ephraim P. Engleman Rheumatology Research Center, Division of Rheumatology, University of California, San Francisco, CA USA

**Keywords:** Scleroderma, Systemic sclerosis, Autoimmune disease, Genetics, Single nucleotide polymorphisms, Genomics, Mouse models, Heterogeneity

## Abstract

Significant advances have been made in understanding the genetic basis of systemic sclerosis (SSc) in recent years. Genomewide association and other large-scale genetic studies have identified 30 largely immunity-related genes which are significantly associated with SSc. We review these studies, along with genomewide expression studies, proteomic studies, genetic mouse models, and insights from rare sclerodermatous diseases. Collectively, these studies have begun to identify pathways that are relevant to SSc pathogenesis. The findings presented in this review illustrate how both genetic and genomic aberrations play important roles in the development of SSc. However, despite these recent discoveries, there remain major gaps between current knowledge of SSc, a unified understanding of pathogenesis, and effective treatment. To this aim, we address the important issue of SSc heterogeneity and discuss how future research needs to address this in order to develop a clearer understanding of this devastating and complex disease.

## Introduction

Systemic sclerosis (SSc, the systemic form of scleroderma) is a complex disease with features that include autoimmunity, vasculopathy, and fibrosis. The disease is more common in women (4:1 female to male ratio) [1, 2]. There is significant clinical heterogeneity between SSc patients which remains poorly understood [3], and there remain critical gaps in understanding of the biologic basis of SSc. The core signs and symptoms of SSc are Raynaud’s phenomenon, skin thickening, and serum autoantibody production, but patients have different patterns of internal organ involvement with variable presentations and outcomes [4, 5]. SSc patients overall have an estimated 66 % 10-year survival rate, which decreases to 38 % for those with significant internal organ involvement [6, 7]. Pulmonary fibrosis and pulmonary artery hypertension (PAH) are leading causes of death and affect approximately 15 % of SSc patients [8]. Cardiac disease including left- and right-sided heart failure, conduction system abnormalities, arrhythmias, or pericardial disease affects 15–35 % of patients [9–12]. Skin involvement causes significant disability and also correlates directly with increased mortality [13]. The presence of cardiac involvement portends a poor prognosis with 70 % 5-year mortality [14] contributing to roughly 25 % of SSc-related deaths [15].

SSc is a rare disease with an estimated 50 to 300 cases per million, and while the overall genetic burden is modest (only 2.6 % of SSc patients’ siblings develop SSc), evidence from familial, twin, and epidemiologic studies has implicated genetic predisposition for disease [16–19]. A positive family history raises relative risk by 15- to 19-fold in siblings relative to the general population, and first-degree relatives also have increased risk for developing Raynaud’s phenomenon and interstitial lung disease [20, 21]. However, the genomic variants identified to date only account for approximately half of the genetic burden of SSc; environmental and epigenetic factors are thought to play a major role in this “missing heritability,” and recent work has implicated multiple different epigenetic risk factors [22–24].

Like most autoimmune diseases, the genetic contribution to scleroderma is not due to a single rare genetic mutation but rather derives from many common genetic variants which predispose patients to disease. As an autoimmune disease, it has been well established that SSc is associated with HLA loci, and these studies have been recently reviewed elsewhere [24, 25]. Through the use of high-throughput technologies including genomewide association studies (GWAS), researchers have identified and confirmed over 25 additional non-HLA SSc-associated genetic loci. The vast majority of these regions overlap with those that have been implicated in other autoimmune diseases. In this review, we will first briefly review this rapidly expanding area and then discuss other approaches that have implicated genetic pathways in SSc in an attempt to better understand disease pathogenesis.

## Immune Genes Implicated by Large-Scale Genetic Studies

Prior to the past 10 years, beyond the HLA region, no clear SSc genetic susceptibility loci had been identified. However, with the advent of advances in genetic technologies and the development of national and multinational case–control cohorts, there have been an increasing number of studies that have identified significant genetic associations with systemic sclerosis. In this review, we will focus on loci that have been identified as genomewide significant and those that have been replicated.

Using genetic association results obtained for other autoimmune diseases including systemic lupus erythematosus and rheumatoid arthritis to identify candidate genes, significant associations have been identified between SSc and single nucleotide polymorphisms in the *BANK1*, *BLK*, *CD226*, *IL2RA*, *IL12RB*, *KCNA5*, *IRF5*, *STAT4*, *TNFAIP3*, *TNFSF4*, and *TLR2* genes [26–40].

**Fig. 1:**
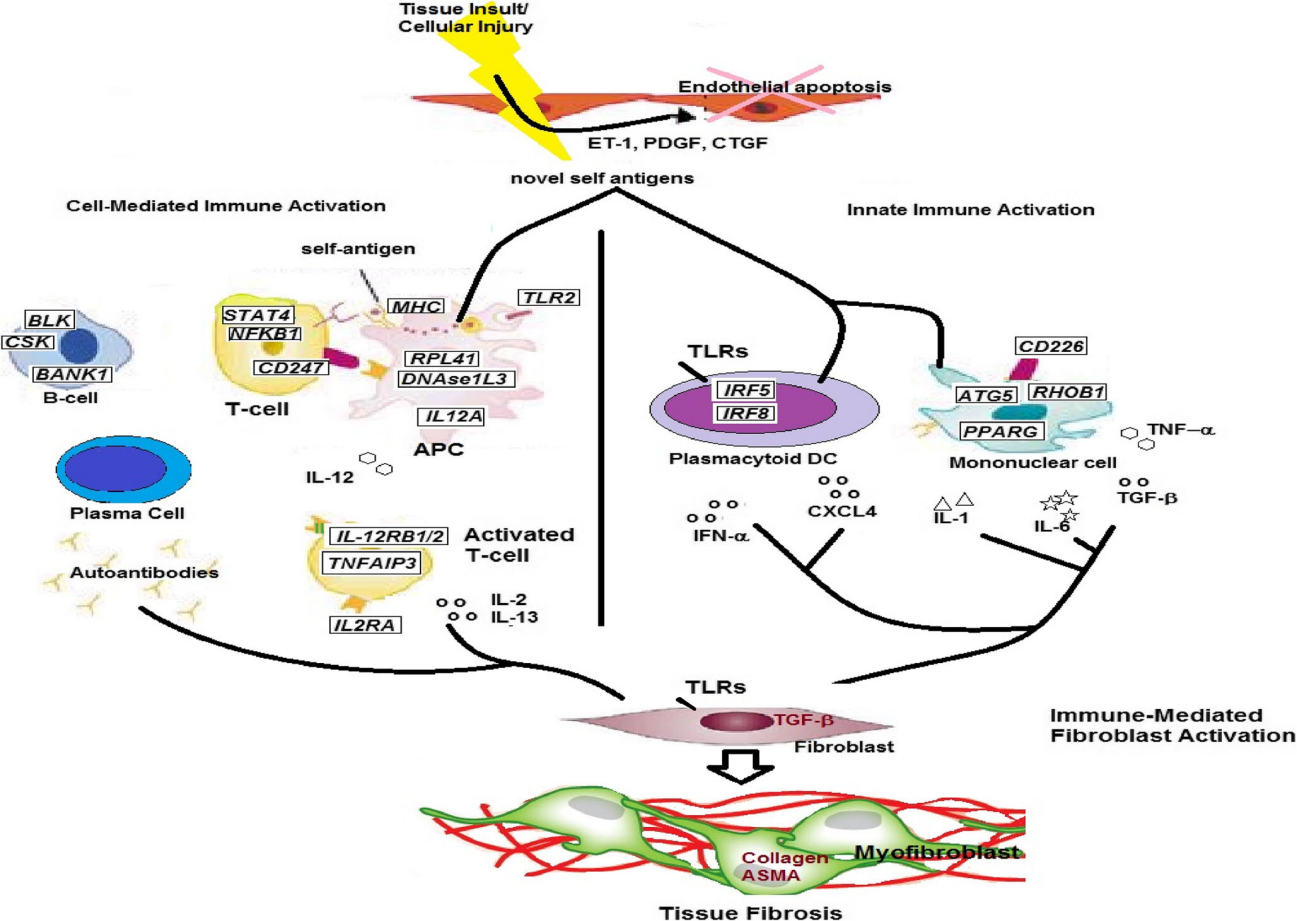
Schematic of cellular roles for molecules genetically implicated in SSc pathogenesis. Tissue injury leads to release of self antigens and subsequent cell-mediated (via MHC) and innate (via TLRs) immune activation. Cells implicated in SSc and molecules genetically implicated in SSc (*italicized*, *boxed*) are emphasized. Cell interaction and cell products lead to immune-mediated fibroblast activation and subsequent tissue fibrosis

The advent of GWAS allowed for confirmation of previously reported associations with the MHC region, *IRF5*, and *STAT4* [41–43], and identified *CD247* as a disease-associated locus [44]. Subsequent GWAS and GWA follow-up studies have identified *IRF8* [43, 44], *PSORS1C1* [45], *IL12RB1* [46, 47], *IL12RB2* [35], and *CSK* [39] loci as genomewide significant. In addition to these loci, at least two studies have confirmed significant association (*p* < 1*10^−4^) at the *TNFAIP3*, *TNFSF4*, *ATG5*, *SCHIP1*-*IL12A*, and *DNASE1L3* loci (Table 1). While the evidence confirming their association is not yet available, studies have now identified an additional 17 loci that have been demonstrated to have associations with SSc (p*10^−4^ > *p* > 5*10^−8^). These associations are summarized in Table 2, while Fig. 1 illustrates how many of these polymorphisms may contribute to disease pathogenesis.

**Table 1:** Confirmed genomewide significant non-HLA associations or studies with two independent replications with *p* < 5*10^−4^

Symbol	Gene name	Locus	SNP	Approach	Case/control	SSc phenotype	OR	*p* value
ATG5 [50]	Autophagy-related 5	6q25	rs9373839	Immunochip	1833/3466	SSc	1.19	3.8*10^−8^
*CD247* [44, 45]	T cell receptor zeta-chain	1q22	rs2056626	GWAS	2296/5171	SSc	0.82	3.4*10^−9^
CSK [39]	c-src	15q24	rs1378942	GWA FU	5270/8326	SSc	1.20	5.0*10^−12^
DNASE1L3 [50]	Deoxyribonuclease I-like 3	3p14	rs35646470	Immunochip	1833/3466	ACA (and all)	2.03	4.3*10^−31^
IL12RB1 [47]	IL-12 receptor beta-1	19p13	rs2305743	GWA FU	8697/5032	SSc	0.81	4.3*10^−10^
IL12RB2 [35]	IL-12 receptor beta-2	1p31	rs3790567	GWA FU	3344/3848	SSc	1.17	2.8*10^−9^
*IRF5* [44, 45]	Interferon response factor 5	7q32	rs10488631	GWAS	2296/5171	SSc	1.49	3.8*10^−14^
IRF8 [64]	Interferon response factor 8	7p12	rs11642873	GWAS	3360/10,143	lcSSc	0.75	2.3*10^−12^
PSORS1C1 [45]	Psoriasis susceptibility 1 candidate 1	6p21	rs3130573	GWAS	564/1776	SSc	1.25	5.7*10^−10^
SCHIP1-IL12A [50]	Schwannomin interacting protein 1/interleukin 12 alpha	3q25	rs77583790	Immunochip	1833/3466	SSc (lcSSc)	2.57	1.2*10^−11^
*STAT4* [44, 45]	Signal Transducer and activator of transcription 4	2q32	rs3821236	GWAS	2296/5171	SSc	1.30	3.9*10^−9^
*TNFAIP3* [28, 37]	TNF-associated interacting protein 3	6q23	rs5029939	CG	1202/1196	SSc	2.08	1.2*10^−7^
*TNFSF4* [9, 31, 93]	TNF superfamily member 4	1q25	rs2205960	CG	1031/1014	ACA+	1.33	1.3*10^−5^

Genes that have been shown to be significant in two or more studies are in italics

*lcSSC* limited cutaneous systemic sclerosis, *SNP* single nucleotide polymorphism, *OR* odds ratio, *CG* candidate gene, *GWAS* genomewide association study, *GWA FU* GWAS follow-up study, *SSc* systemic sclerosis, *ATA* anti-topoisomerase I antibody, *ACA* anti-centromere antibody, *SScPAH* SSc-associated pulmonary arterial hypertension

**Table 2:** Additional SSc genetic associations with one study with *p* value between 5*10^−4^ and 5*10^−8^

Symbol	Gene name	Locus	SNP	Approach	Case/control	SSc phenotype	OR	*p* value
BANK1 [27, 94]	B cell scaffold protein with ankyrin repeats 1	4q24	rs10516487	CG	1295/1137	dcSSc	1.30	4.0*10^−4^
BLK/C8orf13 [32, 85]	B lymphocyte kinase/chromosome 8 open reading frame 13	8p23	rs2736349	CG	1639/1416	SSc	1.27	6.8*10^−5^
CD226 [33]	Cluster of differentiation 226	18q22	rs763361	CG	1990/1642	SSc	1.22	5.7*10^−5^
GRB10 [64]	Growth factor receptor-bound protein 10	7p12	rs12540874	GWAS	3360/10,143	lcSSc	1.15	1.3*10^−6^
IL2RA [40]	IL-2 receptor alpha	10p15	rs2104286	CG	3023/2735	ACA+	1.30	2.1*10^−4^
JAZF1 [51]	JAZF zinc finger 1	7p15	rs1685352	GWAS	2761/3720	SSc	1.14	3.6*10^−5^
KCNA5 [30]	Potassium voltage-gated channel, shaker-related subfamily, member 5	12p13	rs10744676	CG	1576/1033	SScPAH	0.64	3.0*10^−4^
KIAA0319L [51]	KIAA0319L	1p34	rs2275247	GWAS	2761/3720	SSc (lc)	1.46	3.9*10^−6^
NKFB1 [39]	Nuclear factor kappa beta 1	4q24	rs1598859	GWA FU	5270/8326	SSc	1.14	1.0*10^−6^
PPARG [52]	Peroxisome proliferator-activated receptor gamma	3p25	rs310746	GWA FU	2921/6963	SSc	1.25	5.0*10^−7^
PSD3 [39]	Pleckstrin and Sec7 domain-containing 3	8p22	rs10096702	GWA FU	5270/8326	SSc	1.18	3.0*10^−7^
PXK [51]	PX domain-containing serine/threonine kinase	3p14	rs2176082	GWAS	2761/3720	SSc (ACA)	1.21	4.4*10^−7^
RHOB1 [45]	Ras homolog family B	2p24	rs13021401	GWAS	564/1776	SSc	1.21	3.7*10^−6^
RPL41 [64]	Ribosomal protein L41	12q13	rs11171747	GWAS	1699/10,143	dcSSc	1.23	6.0*10^−8^
SOX5 [64]	Sex-determining region Y-box 5	12p12	rs11047102	GWAS	1791/10,143	ACA+	1.36	1.4*10^−7^
TLR2 [36]	Toll-like receptor 2	4q32	rs5743704	CG	1622/1462	SSc	2.24	3.0*10^−4^
TNIP1 [45]	TNFAIP3 interacting protein 1	5q32	rs2233287	GWA FU	4389/7611	SSc	1.19	1.9*10^−4^

While most of these studies have been extensively reviewed elsewhere [48, 49], three new studies in the past year have shed additional insights into the immunogenetics of SSc. In one of the largest genetic studies to date, Mayes et al. genotyped 1833 SSc cases and 3466 controls with the Immunochip, a custom SNP genotyping array that provides high-density mapping of autoimmune disease-associated loci [50]. Using this approach, the authors identified novel associations at the *DNASE1L3*, *SCHIP1*-*IL12A*, and *ATG5* loci [50]. Additionally, this work allowed dense HLA mapping stratified by antibody status (centromere and topoisomerase); using this large collection, and employing imputation and conditional analysis, they were able to identify a model composed of six polymorphic amino acid positions and seven SNPs which explains all observed associations in the HLA region in SSc and its serological subphenotypes. In a second study by Martin et al., the authors performed a meta-analysis of previous GWAS including both SSc and systemic lupus erythematosus (SLE) patients for a total of 6835 cases and 14,274 controls [51]. After replication of top hits in an independent SSc case–control study, this study identified novel SSc associations at *KIAA0319L* and the previously described SLE susceptibility loci *PXK* and *JAZF1*. An additional GWA follow-up study identified a genomewide significant association of SSc with polymorphism near *PPAR*-γ [52], a gene implicated in metabolism, immunity, and protection from fibrosis [53].

## Pathways and Potential Biomarkers Implicated by Human Transcriptomic and Proteomic Studies

While genomic technologies have yielded major insights into genetic predisposition, utilization of other “omics” approaches, including transcriptomics and proteomics, has yielded additional important insights into pathways activated during SSc and in different patient subsets.

### Microarray Intrinsic Subsets

Given the availability of skin as a target tissue in SSc, transcription profiling of skin biopsies from scleroderma patients has been undertaken and has yielded significant insights into scleroderma. Microarray studies have identified pathways that are activated in patients with disease and also identified novel patient subsets based on molecular expression patterns. Beyond the clinical classification of limited and diffuse SSc, the lack of biomarkers to explain patient heterogeneity has been a major limitation in identifying patients with different prognoses, and skin microarray has identified novel ways of evaluating SSc patients. Furthermore, the gene expression pattern has been shown to serve as a genetic biomarker for skin severity as a 44-gene subset can predict patients’ modified Rodnan skin score (MRSS), a validated SSc-specific skin disease severity score [54, 55].

Skin biopsy microarrays are not only able to distinguish SSc from normal skin but also have proven helpful to classify SSc patients into fibroproliferative, inflammatory, limited, and normal-like subsets based on a core set of genes known as an “intrinsic subset” [54]. Michael Whitfield and colleagues have subsequently confirmed the validity of these subsets [55–58] and utilized them to better understand molecular stratification of patients.

The inflammatory group, marked by genes from the immune system response and inflammatory response Gene Ontology pathways, also includes upregulation of interferon-inducible genes, genes involved in vasculature development, and genes associated with fibrosis [54, 55]. This group tends to include patients with aggressive skin disease but more robust response to immunosuppressive treatment (mycophenolate mofetil) [58]. In contrast, the fibroproliferative group expresses genes from the mitosis, chromosome segregation, and DNA metabolic process pathways and tends to be more treatment refractory [54, 55, 58]. While initially thought to be intrinsic and identifying stable disease subsets [55], subsequent studies have shown that individual patients can change subsets both over time and in response to treatment with mycophenolate mofetil [58]. While not the primary pathway identified in unbiased analyses, the TGF-β pathway has been shown to be activated in patients with a fibroproliferative intrinsic subset; this also correlates with downregulation of the PPAR-γ pathway [56, 59].

### Interferon-Inducible Signature

In peripheral blood and PBMCs, microarray studies have identified that roughly half of SSc patients possess an interferon signature similar to that seen in SLE and other autoimmune diseases [60–62]. Other studies have shown that plasmacytoid dendritic cells (pDCs) are the primary source of the interferon [63]. Given this finding, along with the association of SSc with polymorphisms in interferon regulatory factors *IRF5* and *IRF8* [44, 64], the interferon pathway may be playing a critical role in modulating SSc pathogenesis [65]. One study demonstrated that the plasma interferon score was higher in SSc patients than controls and correlated with Medsger disease severity index and pulmonary function parameters [66].

### CXCL4

Proteomic analysis is still in its infancy but holds tremendous promise for the identification of potential biomarkers. In a recent study, proteomewide analysis showed that CXCL4 is the predominant protein secreted by pDCs in SSc, both in circulation and in skin [67]. The levels seen in SSc patients were substantially higher than those seen in other autoimmune diseases such as SLE and ankylosing spondylitis, higher in diffuse cutaneous than limited cutaneous disease, and higher in earlier dcSSc than in long-standing disease. Furthermore, levels correlated with skin and lung fibrosis and with pulmonary arterial hypertension, indicating that this may represent a novel disease-specific biomarker with prognostic significance.

In another study which used proteomics from pDCs to identify novel biomarkers, plasma levels of the Toll-like receptor agonist S100A8/9 were found to be elevated in SSc patients compared to controls [68].

## Insights From Rare Sclerodermatous Diseases

### Cancer-Associated RNA Polymerase III Antibody SSc

Anti-RNA polymerase 3 antibodies are observed in roughly 10 % of SSc patients although prevalence is variable based on genetics and geography [69]. Joseph et al. performed an elegant study to determine whether RNA polymerase III antibodies may derive from cancer among the subset of SSc patients who develop them [70]. In previous studies, RNA pol III patients have been identified as being at a significantly increased risk of cancer and also of having a cancer diagnosis prior to or near the time of SSc diagnosis [71, 72]. Joseph et al. successfully isolated tumor DNA from histologic slides and identified mutations in the *POLR3* gene or loss of heterozygosity in six of eight patients with cancer and RNA polymerase III antibodies and no patients with SSc and cancer with other autoantibodies [70]. Furthermore, immunologic characterization of the CD4+ T cells from patients with *POLR3* tumor mutations demonstrated the presence of T cells reactive to the RPC1 peptide (an RNA polymerase III subunit encoded by *POLR3*) that are patient, peptide, and HLA specific.

### Stiff Skin Syndrome

The stiff skin syndrome (SSS) is a rare Mendelian disorder caused by mutations in the fibrillin gene, the same gene responsible for Marfan syndrome, which is associated with highly elastic connective tissue. Unlike SSc, SSS does not portend other internal organ manifestations and is not associated with autoimmunity or vasculopathy [73]. Gerber et al. [74] attempted to recapitulate this disease utilizing a knock-in strategy to create a strain of mice carrying a mutated fibrillin-1 allele identified in patients with SSS and another mutant mouse strain (D1545E) harboring an integrin mutation predicted to disrupt integrin binding to fibrillin-1. Both mutant mice strains developed dermal fibrosis accompanied by excess collagen deposition as well as progressive loss of intradermal adipose tissue. In addition to skin fibrosis, the transgenic mice spontaneously developed marked cutaneous inflammation, with accumulation of pDCs, Th2- and Th17-skewed T helper cells, and plasma cells. Moreover, these mice developed circulating anti-topoisomerase I antibodies. Because fibrillin-1 is known to modulate TGF-β signaling and because the SSS fibrillin-1 mutations specifically affect the integrin-binding domain, the authors speculated that the stiff skin phenotype might be due to unchecked TGF-β activation and increased TGF-β signaling [74]. Interestingly, treatment of the mutant mice with a neutralizing antibody to TGF-β, as well as alpha-1 integrin-activating antibody, was able to reverse the fibrotic process and mitigate the immune dysregulation.

## Insights From Genetic Mouse Models of Scleroderma

The most commonly utilized mouse model of scleroderma remains the bleomycin model which nicely recapitulates many of the seminal features of the disease, including fibrosis and inflammation. However, because this is a chemical injury model with largely unknown molecular mechanism(s), multiple attempts have been made to engineer or discover genetic mouse models that may yield important insights into disease pathogenesis. While none of the genetic models to date adequately recapitulates all disease features, the growing diversity of models enables researchers to study different aspects of disease and determine the effects of modulation of multiple relevant pathways on outcomes important in SSc. In Table 3, we summarize 11 different genetic models which appear to recapitulate important aspects of SSc.

**Table 3:** Genetic mouse models of scleroderma

Model	Gene	Cell-specific ablation	Pathway	Organs	Fibrosis	Vasculopathy	Inflammation	Autoimmunity
TSK1 [95]	Fibrillin-1	–	TGF-beta	Skin (hypodermis)	Y	Y	N	Y
Tsk2 [96]	Chr1, gene not currently known	–	TGF-beta	Skin	Y	Y	Y	Y
SSS mouse [74]	Fibrillin-1	–	TGF-beta	Skin	Y	Y	Y	Y
TBRICA; Cr-ER [79]	TGF-beta receptor	Fibroblast	TGF-beta	Skin, lung, kidney, vasculature	Y	Y	N	N
TBRIIdk [102]	TGF-beta receptor	Fibroblast	TGF-beta	Skin, lung	Some	Some	Y	N
CTGF Tg [97]	CTGF	Fibroblast	TGF-beta	Skin, lung, kidney, vasculature	Y	Y	N	N
Caveolin KO [98]	CAV1	–	–	Lung, heart, pulmonary artery	Y	Y	N	N
Fli1 [99]	FLI1	–	–	Skin	Y	Y	N	N
Fra-2 [101]	Fos-related protein 2	–	PDGF	Skin, pulmonary artery, vasculature	Y	Y	–	–
sUPAR [87]	Soluble urokinase-type plasminogen activator receptor	–	UPA/plasmin	Skin, lung	Y	Y	N	–
Wnt-10b [82]	Wnt-10b	Adipose	Wnt	Skin	Y	N	N	–

### Tsk1 and Stiff Skin Syndrome Models: Fibrillin-1

The tight skin mouse, which shows prominent spontaneous fibrosis of the hypodermis, has been widely used as a genetic mouse model of SSc, although exactly how this model corresponds with human disease has been difficult to explain. The Tsk1 mouse has now been shown to be caused by mutations in the fibrillin gene [75–77]. In the recent model by Gerber et al. discussed in “Stiff Skin Syndrome,” knock-in human fibrillin-1 mutations were introduced and the mice demonstrated a phenotype that included dermal fibrosis, inflammation, and autoimmunity. It is interesting that previous descriptions of human SSS have not described either a significant inflammatory component in the skin lesions nor described the autoimmunity that is seen in the mice. Whether this represents poor clinical characterization of human SSS due to its rarity or whether the mouse model more closely resembles SSc than SSS, the mouse model clearly recapitulates aspects of human fibrotic skin disease. It is not clear, however, whether it is a model of SSS, SSc, or a unique murine entity that lacks a human correlate.

### Tsk2

The Tsk2 mouse may more closely mimic human disease (it spontaneously displays fibrosis, inflammation, and autoimmunity); while the ENU-induced locus is on chromosome 1, genetic studies are ongoing to elucidate the genetic lesion in this model [78].

### TGF-β pathway

TGF-β is widely cited as the pathogenic molecule in SSc and other forms of fibrosis. Mice with an inducible constitutively active TGF-β receptor I (TGF-β RI) mutation driven by a fibroblast-specific promoter have been generated and demonstrate fibrosis of the dermis and fibrotic thickening of small blood vessel walls in the lung and kidney [79]. Primary skin fibroblasts from these mice showed elevated expression of downstream TGF-β targets, reproducing the hallmark biochemical phenotype of explanted SSc dermal fibroblasts. Constitutive activation of TGF-β in fibroblasts is therefore sufficient to induce a fibrotic phenotype.

### Wnt and PPAR-γ pathways

The Wnt pathway is a key developmental and homeostatic pathway in multiple tissues, and alterations in the pathway have been shown to be pro-fibrotic. Patients with SSc have increased fibroblast levels of β-catenin, a key Wnt mediator. Mice with fibroblast-specific β-catenin ablation rapidly develop skin fibrosis [80], while pharmacologic treatment with Wnt antagonists can reverse skin fibrosis [81]. Transgenic mice expressing Wnt-10b in adipose tissue showed not only progressive loss of subcutaneous adipose tissue but also dermal fibrosis, increased collagen deposition, fibroblast activation, and myofibroblast accumulation [82]. Wnt activity correlated with collagen gene expression in these biopsy specimens. This suggests that Wnt-10b switches differentiation of mesenchymal cells toward myofibroblasts by inducing a fibrogenic transcriptional program while suppressing adipogenesis.

Fibroblast-specific deletion of PPAR-γ, the master regulator of adipogenesis, results in enhanced susceptibility to bleomycin-induced skin fibrosis and enhanced sensitivity of fibroblasts to TGF-β1 in PPAR-γ-deficient mice. These results indicate that PPAR-γ suppresses fibrogenesis [83].

### Fra-2 and sUPAR: Models of SSc Vasculopathy

The expression of the transcription factor Fra-2 is upregulated in SSc patients and in different mouse models of SSc. Fra-2 transgenic mice have been shown to develop spontaneous skin and lung fibrosis and vasculopathy (including skin microvasculopathy and pulmonary artery enlargement consistent with pulmonary hypertension) which are mediated by TGF-β and PDGF [84]. This is particularly important because most other mouse models (both genetic and inducible) do not demonstrate significant vasculopathy which is a key clinical feature of SSc [86].

Urokinase-type plasminogen activator receptor (uPAR) has been implicated in SSc microvasculopathy. Mice deficient in soluble uPAR demonstrate skin fibrosis as well as decreased microvessels which were shown to have undergone apoptosis [87]. While these mice did demonstrate lung disease, it was more reminiscent of chronic pneumonia than of pulmonary fibrosis.

## SSc Heterogeneity and Genetics

Nearly all of the genetic associations reviewed in “Immune Genes Implicated by Large-Scale Genetic Studies” represent genes involved in immunity, and associations with the same genes have been reported in multiple other autoimmune diseases. Despite the prominent fibrotic features of SSc, genetic studies to date have not identified major risk factors related to genes involved in the process of fibrosis [88]. Interestingly, recent studies have looked at SSc lung disease and idiopathic pulmonary fibrosis (IPF) and found little genetic overlap, suggesting that the fibrosis in SSc and ILD may be distinct [89, 90]. One explanation for the lack of fibrotic genes identified may be that the design of many of the studies, including the recent Immunochip and SSc–SLE pan-meta-GWAS, was biased toward identification of immune system loci. Because a core set of known fibrotic genes has not yet been identified, a similar strategy to enhance the identification of fibrotic genes is not as easily achievable. Furthermore, if fibrosis is secondary to either epigenetic changes or a process secondary to an aberrant immune response (see Fig. 1), that could explain the lack of fibrotic genes identified in otherwise largely agnostic GWAS.

Identification of genetic variants that contribute to SSc has been complicated by the complexity and heterogeneity of the disease. SSc behaves more like a syndrome than a unified disease as SSc patients can have multiple different clinical phenotypes and patterns of organ involvement. As personalized medicine advances, we may learn that there are in fact multiple or even numerous unique disease entities that are all currently classified as SSc. Cancer-associated RNA polymerase III antibody-associated SSc [70] is probably one example of molecularly defined disease that has a novel pathogenesis and may respond differently to treatment. For the purpose of understanding disease, heterogeneity likely hampers scientists’ ability to identify genetic risk loci because cases represent patients with a multitude of SSc-related conditions.

The contribution of race/ethnicity represents another complicating factor; there are genetic differences between and across ethnicities which affect prevalence and relative importance of genetic susceptibility loci. Furthermore, Caucasian and Asian populations have been well represented in genetic studies compared to African-Americans, who have more severe SSc manifestations. Indeed, all of the large-scale genetic studies reviewed in “Immune Genes Implicated by Large-Scale Genetic Studies” have focused on Caucasian populations, and further analysis of other ethnic groups may yield new genetic insights, as has been seen in other diseases [91].

Attempts to subclassify patients are frequently made on the basis of skin disease (limited versus diffuse cutaneous disease), autoantibodies, and organ involvement (particularly lung disease [3, 92]). There has already been substantial work to determine whether genetic associations are present only in certain SSc subtypes (mostly lcSSc/dcSSc and ATA/ACA). However, limited clinical phenotyping makes it difficult to study homogeneous SSc patient groups that represent disease endophenotypes and may have more distinct and clear-cut genetic predispositions.

More recently, the use of skin biopsy microarray intrinsic subsets as a way of classifying disease has been proposed as identifying disease endophenotypes [92]. Identification of additional novel biomarkers may further enable biological classification into subtypes, and that should hopefully contribute to better understanding of patients’ genetic susceptibility to SSc. With functional studies such as gene expression analysis, another challenge for understanding SSc is studying specific classes of SSc cells and tissues. Because SSc has multiple diverse manifestations, it is difficult to determine which cell type is most appropriate for investigation. A great deal of focus has been on dermal fibroblasts, but whether these cells are primary or secondary in disease pathogenesis remains unclear. Similarly, while immune cells are clearly relevant, it remains unclear whether lymphocytes, dendritic cells, macrophages, or other cell types are most relevant. While the vasculature and lungs are clearly important, these tissues remain difficult to acquire. Skin biopsy remains an important tissue which is accessible and is of diagnostic/prognostic significance, but because skin is very heterogeneous (keratinocytes, dermal fibroblasts, immune cells, adipocytes, vessels), analysis of gene expression may be obscured by genetic “noise” from multiple cell types. As cell-based assays and systems biology approaches develop further, it will be increasingly important to identify which cells to study and to study them in isolation in order to understand the biological relevance of SSc genetic associations. Figure 1 illustrates which cell types the identified genetic variants affect and how these may contribute to the pathogenesis of SSc.

With the advent of next-generation sequencing, there is also a movement toward personalized genomics. Despite the recent identification of a number of susceptibility loci in SSc as reviewed in “Immune Genes Implicated by Large-Scale Genetic Studies,” other than HLA, the variants identified are not likely to be the causative variants. This is because GWAS utilize common (with the minor allele >5 % in the population) single nucleotide polymorphisms that are in linkage disequilibrium with implicated causative genetic variants. Furthermore, the total contribution of susceptibility loci discovered by GWA and follow-up studies explains only a fraction of the “heritability” of the disease. These concepts lead many to believe that there exist many rare variants with relatively large effects, which, in aggregate, account for the remaining prevalence of the disorder. These so-called private mutations can be identified with exome or whole genome sequencing and may be able to explain individual patients’ genetic predisposition for disease. While such private mutations may be present in only one or very few affected individuals, identification of multiple private mutations within genes or in common pathways may also help elucidate disease pathogenesis that is more broadly relevant and inform the development of therapies that may help classes of patients.

While genetics clearly contributes importantly to SSc pathogenesis, it does not fully explain disease susceptibility. Environmental factors and associated epigenetic changes likely contribute importantly as well [23]. In addition to better epidemiologic studies to identify exposures such as chemicals and viruses that may influence disease risk, studies of epigenetic DNA modifications may also be crucial to understanding the molecular processes central to SSc.

SSc remains one of the most mysterious and difficult to treat diseases in modern medicine. While many studies have provided novel insights into genetic risk factors and pathways that are dysregulated, there remains a huge gap between current knowledge, understanding of pathogenesis, and the identification of effective treatments. The heterogeneity of SSc complicates its understanding, but modern omics technologies and better clinical phenotyping are contributing toward the goal of SSc being a disease that can be understood with systems biology tools and more effectively treated with a personalized medicine approach.
